# Preparation of HfC_x_N_1−x_ Nanoparticles Derived from a Multifunction Precursor with Hf-O and Hf-N Bonds

**DOI:** 10.3390/ma16124426

**Published:** 2023-06-16

**Authors:** Guang Zeng, Ping Xu, Chen Zeng, Qizhong Huang, Zhean Su

**Affiliations:** National Key Laboratory of Science and Technology on High-Strength Structural Materials, Central South University, Changsha 410083, Chinaqzhuang@csu.edu.cn (Q.H.)

**Keywords:** HfC_x_N_1−x_, pyrolysis, ultra-high-temperature ceramics, nanoparticles

## Abstract

HfC_x_N_1−x_ nanoparticles were synthesized using the urea-glass route, employing hafnium chloride, urea, and methanol as raw materials. The synthesis process, polymer-to-ceramic conversion, microstructure, and phase evolution of HfC_x_N_1−x_/C nanoparticles were thoroughly investigated across a wide range of molar ratios between the nitrogen source and the hafnium source. Upon annealing at 1600 °C, all precursors demonstrated remarkable translatability to HfC_x_N_1−x_ ceramics. Under high nitrogen source ratios, the precursor exhibited complete transformation into HfC_x_N_1−x_ nanoparticles at 1200 °C, with no observed presence of oxidation phases. In comparison to HfO_2_, the carbothermal reaction of HfN with C significantly reduced the preparation temperature required for HfC. By increasing the urea content in the precursor, the carbon content of the pyrolyzed products increased, leading to a substantial decrease in the electrical conductivity of HfC_x_N_1−x_/C nanoparticle powders. Notably, as the urea content in the precursor increased, a significant decrease in average electrical conductivity values was observed for the R4-1600, R8-1600, R12-1600, and R16-1600 nanoparticles measured at a pressure of 18 MPa, yielding values of 225.5, 59.1, 44.8, and 46.0 S·cm^−1^, respectively.

## 1. Introduction

Hafnium carbide (HfC) has garnered significant attention as a promising material for thermal protection applications due to its exceptional properties such as a high melting point, hardness, and thermal and chemical resistance [1,2,3]. Furthermore, recent studies have unveiled additional potential applications of HfC in catalysis [4,5,6], microwave absorption [7,8], and batteries [9,10]. These emerging fields impose more stringent demands on the particle size of HfC, emphasizing the need for precise control in this aspect.

Material properties are affected by the way it is prepared. Currently, HfC can be prepared by different methods, including the direct carbon-Hf reaction or carbothermal reduction within a pressure-less sintering process [11,12], chemical vapor deposition processes [13,14,15], the sol-gel method [16], and the polymer-derived ceramics (PDC) method [17,18]. Even though each has been used successfully, the PDC method is the technology that can be applied in the broadest range of environments. The preparation of UHTC materials from this method can be tailored to the preceramic precursor at the molecular level to obtain unique chemical/phase compositions and microstructures. There are three categories to design the precursor for preparing HfC ceramics, including Hf-C [19,20], Hf-O [17,21], and Hf-N [18,22]. Among them, the Hf-O bonds method shows the best cost and efficiency advantages. The transformation of these precursors occurs during ceramization, which initially transforms them into HfO_2_, then to HfC with a multi-porous structure when temperatures exceed 1600 °C. The high reduction temperature and porous microstructures, however, make these materials restricted for wide applications. Conversely, precursors with Hf-C or Hf-N bonds could be able to be transformed at relatively low temperatures, which produced ceramics with enhanced high-temperature properties. In contrast to Hf-N bonds, the process of generating Hf-C bonds, reliant on Grignard reagents or n-butyllithium, poses much harsher conditions, thus greatly limiting the application of Hf-C bond methods. Meanwhile, the introduction of N atoms to the precursor improves the densification of ceramics and reduces the conversion temperature of HfC [23].

Currently, research in related fields has the following challenges: On the one hand, the synthesis of precursors based on both Hf-O and Hf-N bonds has only been described in a few papers. On the other hand, the amount of nitrogen source used to modify Hf-N bonds was relatively small, and a higher ratio has not been achieved.

Herein, a precursor with Hf-O and Hf-N bonds was synthesized and converted to an HfC_x_N_1−x_ nanoparticle by HfCl_4_ and urea/methanol. This study considered a wide range of nitrogen source/hafnium source molar ratios much higher than previous studies. The content of nitrogen in HfC_x_N_1−x_ was well controlled by adjusting the annealing temperature and nitrogen source rate. We provide a new route for the preparation of hafnium carbide by introducing a larger proportion of nitrogen, so that the product is hafnium nitride rather than hafnium oxide at lower temperatures, and as the temperature increases, hafnium nitride reacts with carbon in a carbothermal reduction reaction to eventually obtain hafnium carbide. With a high value of R, HfC can be prepared in a new way. HfN is produced instead of HfO_2_ at lower temperatures, and as the temperature increases, HfN reacts with carbon to produce HfC ceramics. The synthesis process, polymer-to-ceramic transformation, and effect of nitrogen contents were described and studied. It has been found that the urea ratio has an effect on the content of carbon in ceramic products, and then affects their electrical conductivity. Further details and results will be presented below.

## 2. Experimental

### 2.1. Materials

Hafnium chloride (HfCl_4_, AR, 99.5%, Sigma Aldrich, Waltham, MA, USA) and urea (CH_4_ON_2_, AR, 97%, Aladdin, Shanghai, China), as well as methanol (CH_4_O, AR, 99%, Aladdin), were purchased and used as received. All the chemical reagents were of analytical grade without further purification.

### 2.2. Synthesis of Precursors

**Figure 1:**
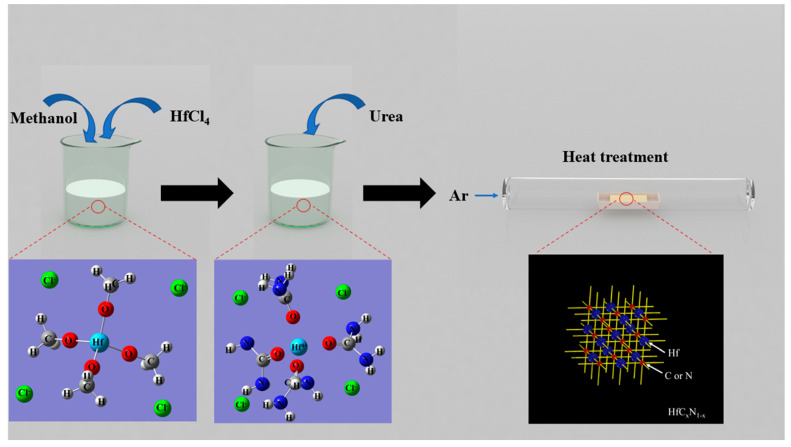
Schematic illustration of the synthesis of HfC_x_N_1−x_ nanoparticle.

The HfC_x_N_1−x_ precursor was synthesized using HfCl_4_, urea, and methanol as the source materials. The preparation process is illustrated in Figure 1. Initially, 3.20 g of HfCl_4_ powder was dispersed in 20 mL of methanol to achieve the desired concentration (0.5 M), resulting in a stable and clear solution. Subsequently, urea was added to the solvent and stirred until complete dissolution. To obtain the precursors, the urea content in the feedstock was carefully controlled, leading to four distinct formulations. The molar ratios of HfCl_4_ to urea were set as 1:4, 1:8, 1:12, and 1:16, respectively. These precursors were labeled as R4, R8, R12, and R16. Table 1 shows the raw material formulation of each precursor. The resulting mixture was stirred to obtain a clear, viscous, and transparent solution. Subsequently, the precursors were subjected to drying in a vacuum oven. The dried samples were then subjected to thermal treatment under an argon (Ar) atmosphere, gradually increasing the temperature at a rate of 10 °C/min, up to a range of 1000–1600 °C for a duration of 3 h. After the thermal treatment, the samples were cooled to room temperature. The resulting product was obtained as a fine powder, exhibiting a light grey color for samples treated up to 1000 °C and black color for samples treated up to 1400 °C.

### 2.3. Characterization Methods

Fourier transform infrared spectroscopy (FT-IR) and thermal gravimetric and differential scanning calorimetry analysis (TG-DTG) were used to characterize the chemical composition and ceramic yield of the precursor. X-ray diffraction (XRD), elemental analysis, X-ray photoelectron spectroscopy (XPS), and transmission electron microscopy (TEM) were used to characterize the chemical composition and microstructure of the as-prepared ceramics. FT-IR spectra were recorded from 4000–400 cm^−1^ on a spectrometer (Nicolet 6700, Waltham, MA, USA). The TG-DTG analysis was performed using a Mettler-Toledo TGA2 instrument. The XRD patterns were recorded by a powder diffractometer (Rigaku D/Max 2500 VB, Tokyo, Japan) using a Cu/Kα radiation source. The carbon, nitrogen, and oxygen contents of the nanoparticles were measured by a Horiba carbon/sulfur (LECO, CS600, St. Joseph, MI, USA) and a Horiba oxygen/nitrogen (LECO, TCH600, USA) analyzer. XPS studies were conducted on the ThermoFisher instrument (ThermoFisher Escalab 250, Waltham, MA, USA) with Al/Kα irradiation with carbon as an internal standard (C1s = 284.8 eV). TEM studies including mapping analysis were conducted on ground powder samples using a ThermoFisher microscope (Titan G2 60-300, FEI, Hillsboro, OR, USA) coupled with an electron-diffraction spectroscope (EDS). The electrical conductivity of powders at 25 °C was measured using a four-point probe arrangement with pressure control (Jingge, ST2722, Jiangsu, China).

## 3. Results and Discussion

**Figure 2:**
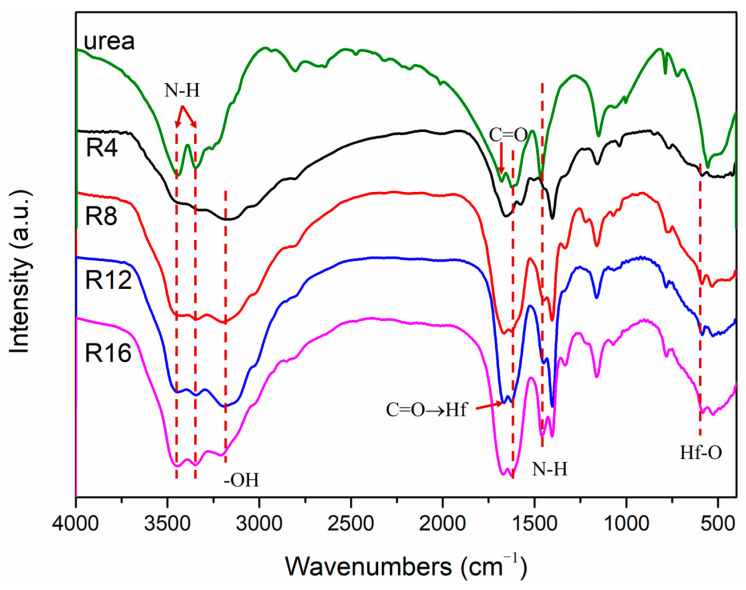
FT-IR spectra of urea and the precursors.

FT-IR provides a wealth of information about the chemical structure of all prepared precursors and urea, as depicted in Figure 2. The presence of -OH stretching vibrations at 3200 cm^−1^ indicates the reaction between HfCl_4_ and methanol. The precursor samples also contained most of the urea vibration peaks. The peak at 1472 cm^−1^ is the N-H bending vibration, and the peak at 3300–3500 cm^−1^ is the N-H stretch vibration [24]. Moreover, Hf-O bond to vibrate at 610 cm^−1^ also could be noticed in Figure 2 [24]. Upon the reaction between the hafnium chloride and urea, the C=O bond undergoes a noticeable shift from 1622 cm^−1^ to 1575 cm^−1^ due to the formation of Hf → O=C [6]. Nevertheless, as the molar ratio of urea/HfCl_4_ increases, the C=O bonds return to 1622 cm^−1^. This is caused by the complexation reaction between the chloride salt and urea. Nevertheless, as more urea is employed, a portion of it remains uninvolved in complexation, existing as a mixture in the precursor.

**Figure 3:**
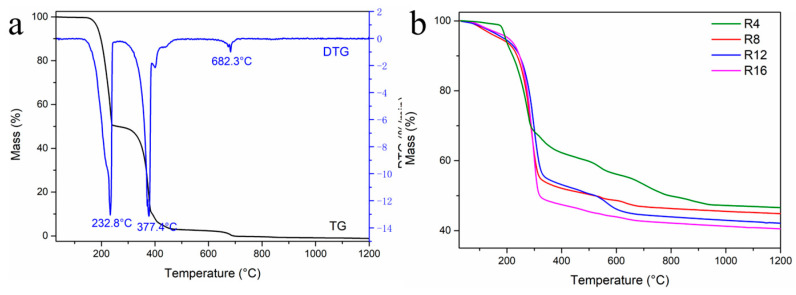
TG-DTG curve of urea (**a**) and TG curves of the ceramization process of precursors (**b**).

The thermal behavior of the precursors during ceramization was evaluated using thermogravimetric analysis (TGA) under a nitrogen atmosphere with a heating rate of 10 °C/min. The results were presented in Figure 3. In Figure 3a, it could be observed that the yield of urea was 0 wt%. Based on the derivative thermogravimetry (DTG) results, a significant mass reduction occurred at 232 °C and 377 °C, corresponding to the boiling and chemical conversion of urea, respectively. As the temperature increases further, there was a slight additional weight loss, and the mass reaches almost zero. The ceramic yields of the R4, R8, R12, and R16 precursors at 1200 °C were 46.5%, 44.8%, 42.1%, and 40.5%, respectively. The weight loss regions observed at 200–300 °C and 300–600 °C might be caused by residual urea and the release of volatile gases such as CH_4_ and NH_3_. The ceramic yields of the precursors decrease slightly with an increase in urea content. According to the TGA results, this type of precursor demonstrates favorable ceramic yield characteristics.

**Figure 4:**
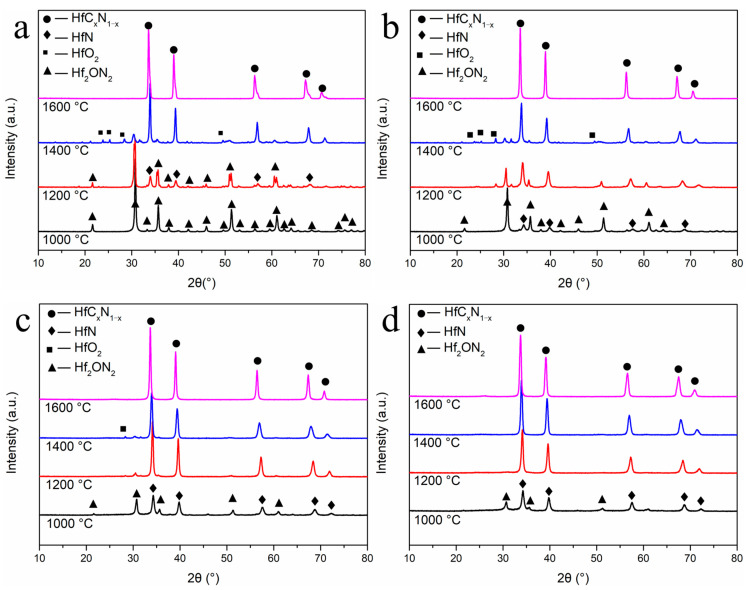
XRD pattern of the precursors pyrolyzed and annealed at different temperatures. (**a**) R4, (**b**) R8, (**c**) R12, (**d**) R16.

X-ray diffraction (XRD) analysis was used to determine the phase composition of all prepared samples. After annealing at 1000 °C, the R4 sample exhibited a single phase of Hf_2_ON_2_, as depicted in Figure 4a. Upon further increasing the annealing temperature to 1200 °C, Hf_2_ON_2_ peaks became more prominent, and a secondary phase of HfN could be observed. As the annealing temperature reached 1400 °C, the crystalline phases of HfN (PDF#97-065-8397) and Hf_2_ON_2_ (PDF#04-007-0073) were gradually consumed and replaced by the HfC_x_N_1−x_ phase (specifically, HfC_0.57_N_0.43_). At 1600 °C, with the release of N_2_ from HfC_x_N_1−x_ (as depicted in Equation (4)), the prepared ceramic exhibited peaks closely resembling the HfC phase. The phase composition was calculated as HfC_0.90_N_0.10_. By applying Vegard’s law, the chemical composition of the HfC_x_N_1−x_ phase was determined based on the lattice parameters of the obtained ceramics. The nitrogen content of HfC_x_N_1−x_ is detailed in Table 1. The average crystalline size of HfC_x_N_1−x_ nanoparticles annealed at different temperatures was calculated using the Scherrer formula, utilizing the half-value width of the (111) peak of HfC_x_N_1−x_. The results are summarized in Table 2.

**Figure 5:**
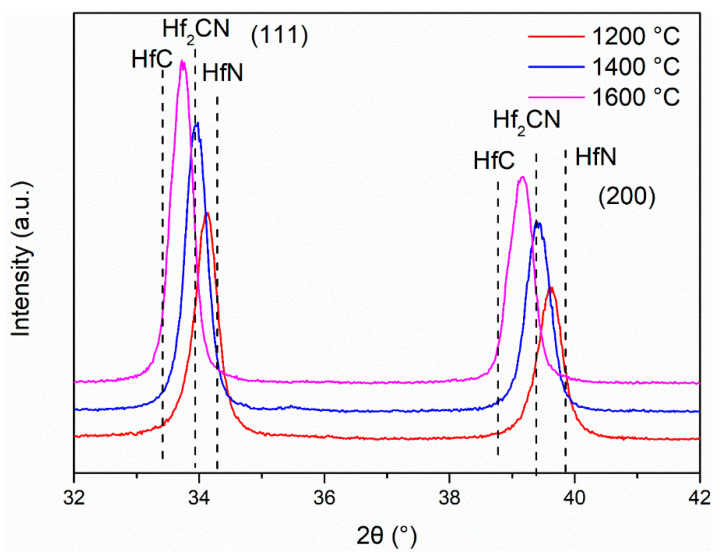
XRD pattern of R16 with the temperature from 1200 to 1600 °C.

There were also obvious differences in phase composition after treatment at the same temperature for samples with different ratios, as shown in Figure 4. At 1000 °C, the HfN phase acted as the secondary phase in the R8 sample (i.e., Hf_2_ON_2_ as the main phase) and as the main phase in the R12 and R16 sample (i.e., Hf_2_ON_2_ as the second phase). It was interesting to see that after heat treatment at 1200 °C, a sharpening of the HfN peak was observed after increasing the nitrogen source content, as well as the disappearance of Hf_2_ON_2_. This was the only phase in the R16 sample to form as close to the HfN phase, without evidence of Hf_2_ON_2_ or HfO_2_ (PDF#00-043-1017). When the proportion of the nitrogen source of the precursor is high, the heat treatment process of the precursor occurs in a nitrogen-rich environment. HfN can be obtained at a lower temperature. With the carbothermal reduction reaction with C and HfN, the precursor can be transformed into HfC_x_N_1−x_ nanoparticles at 1200 °C. By Vegard’s law, the phase composition is calculated as HfC_0.19_N_0.81_. With the annealing temperature increasing, the final phase composition was close to HfC at 1600 °C. More details on the peak shift of HfC_x_N_1−x_ can be found in Figure 5. According to the results of phase composition, the following are the possible chemical reactions:

Hf_2_ON_2_ + C → 2HfN + CO (1)

Hf_2_ON_2_ + 1.75C → 0.25HfO_2_ + 1.75HfC + N_2_ (2)

HfN + xC → HfC_x_N_1−x_ + 0.5xN_2_ (3)

HfC_x_N_1−x_ + C → HfC_x_N_1−x_ + N_2_ (4)

XRD patterns revealed that at 1600 °C, the pyrolyzed samples of R4, R8, R12, and R16 exhibited HfC_x_N_1−x_ as the sole phase. This suggests that all four ratios of samples can be utilized as precursors for HfC. Moreover, an increase in the amount of urea in the precursor led to higher nitrogen content in HfC_x_N_1−x_ after heat treatment at the same temperature. The phase analysis of our precursors demonstrates the ability to regulate the nitrogen content in HfC_x_N_1−x_ ceramics through the manipulation of raw materials and temperature. Additionally, the use of our precursor enables the preparation of HfC_x_N_1−x_ at a relatively low temperature, specifically 1200 °C.

**Figure 6:**
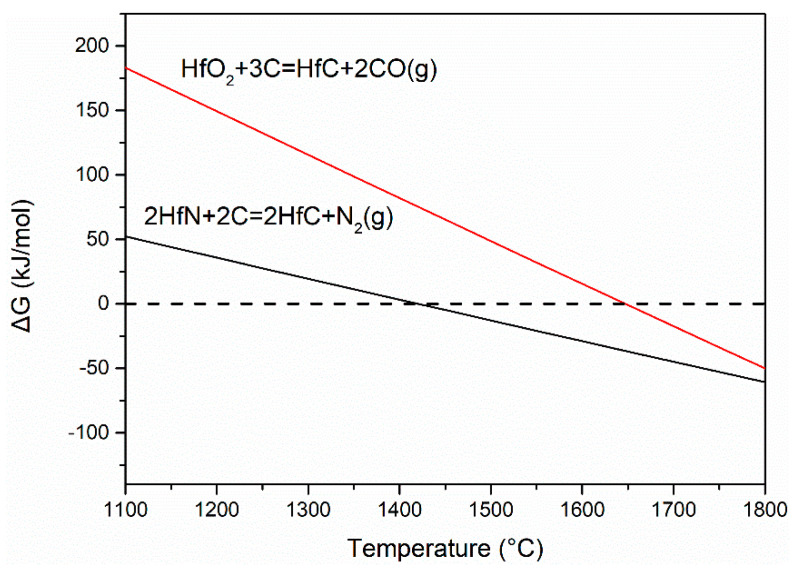
Gibbs free energy of carbothermal reactions versus temperature.

To better explain the decrease in the formation temperature of HfC, the thermodynamic study of carbothermal reaction was conducted using HSC Chemistry. HfC produced by the carbothermal reaction between C and HfO_2_ requires a higher temperature than HfC produced by the carbothermal reaction between C and HfN, as shown in Figure 6 (the Gibbs free energy ΔG of reactions). Upon increasing the urea content, the HfN phase content in the as-prepared ceramic increases after annealing at 1000 °C. With the HfN phase present, HfC can be prepared at a lower temperature. After higher annealing temperatures, solid-solution products formed by HfC and HfN can be obtained.

**Figure 7:**
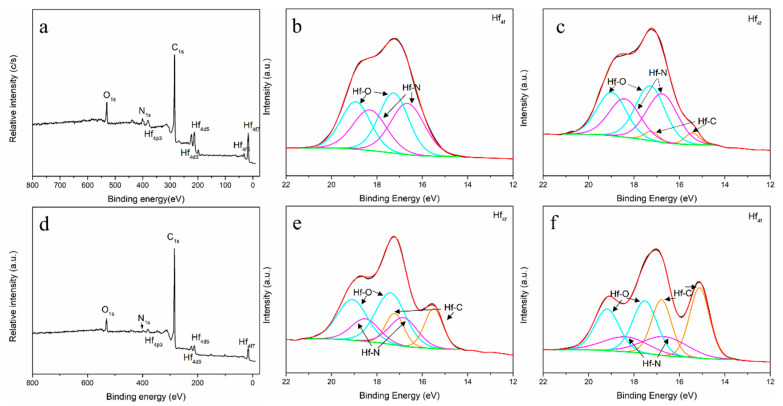
XPS survey spectra (**a**) R16-1000 and (**d**) R16-1600, and the fitted spectra of Hf_4f_ with different temperatures: (**b**) 1000 °C, (**c**) 1200 °C, (**e**) 1400 °C, and (**f**) 1600 °C.

To better understand the fundamental aspects of HfC_x_N_1−x_ formation, XPS was utilized to characterize sample R16 after heat treatment, as shown in Figure 7. In Figure 7a, the XPS spectra recorded for the ceramic of R16-1000 show Hf, C, N, and O present. After heating at 1600 °C, the element contents were almost the same, compared to R16-1600, in Figure 7d. The only difference is that the peak N intensity is significantly reduced. This is caused by the escape of the N atom after heat treatment. In Figure 7b, the Hf4f feature is fit with four peaks, accounting for the 4f 7/2 and 5/2 doublets for Hf-O [25] (at 17.30 eV and 18.97 eV) and Hf-N [5] bond (at 16.76 eV and 18.42 eV) energies. The doublet peaks were fit in Avantage 5.9 software maintaining a standard 1.70 ± 0.1 energy difference and a 0.79 peak area ratio between the 7/2 and 5/2 peaks, following the standard procedure as described previously. As the heat treatment temperature increases to 1200 °C, it is observed that Hf-C bonds are detected at 15.50 eV for the 7/2 peak and 17.23 eV for the 5/2 peak [26], which indicates that HfC_x_N_1−x_ first appears at this temperature. At 1400 °C, it is noted that Hf-O bonds decreased in peak intensity and the intensity of Hf-C bonds increased. The peak intensity of Hf-O decreases further after annealing at 1600 °C, while the peak intensity of Hf-C increases. The existence of the Hf-N bond in the Hf4f spectrum indicated that nitrogen is retained in the ceramics after annealing at 1600 °C. Based on XPS and XRD results, it can be concluded that the ceramics are made up of HfC_x_N_1−x_. According to the literature, the reason for the Hf-O bond exists in Hf4f spectroscopy was exposure to air, resulting in the formation of a thin oxide surface layer [6].

**Figure 8:**
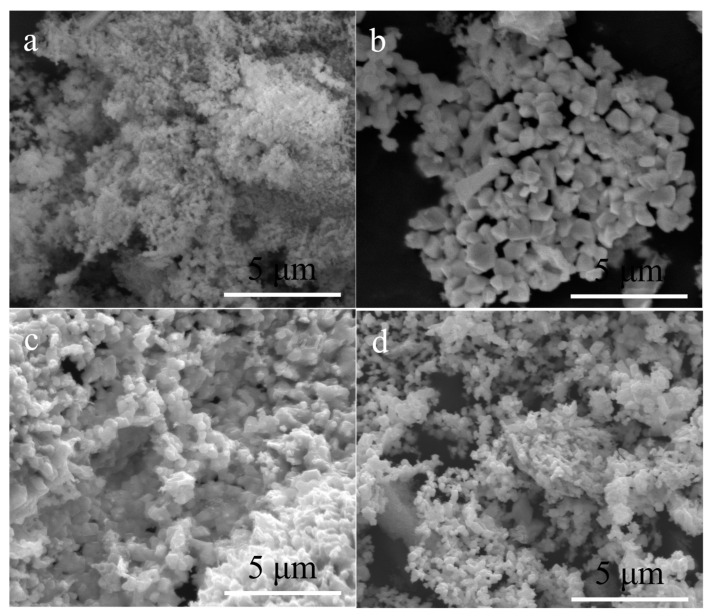
SEM images of R16 treated at different temperatures. (**a**) 1000 °C, (**b**) 1200 °C, (**c**) 1400 °C, (**d**) 1600 °C.

To investigate the sample’s morphology, larger-scale homogeneity, and particle dimensions, SEM analysis was performed as shown in Figure 8. After annealing at 1000 °C, the as-prepared ceramics transform, resulting in the formation of nanoscale HfN and Hf_2_ON_2_ phases. However, during the pyrolysis process, the release of carbonaceous species leads to the deposition of amorphous carbon coatings on the surface of the ceramic particles. These carbon coatings can cause the boundaries between individual particles to appear blurred. When the pyrolysis temperature is increased to 1200 °C, the ceramic particles become more defined. In addition, the growth of HfN grains leads to an increase in the size of the ceramic particles. As the heat treatment temperature continues to rise, the ceramic particle size gradually decreases due to the carbothermal reduction reaction taking place. In Figure 8d, following heat treatment at 1600 °C, the precursors transformed HfC_x_N_1−x_/C nanoparticles, resulting in particle sizes ranging from approximately 80 to 100 nm.

**Figure 9:**
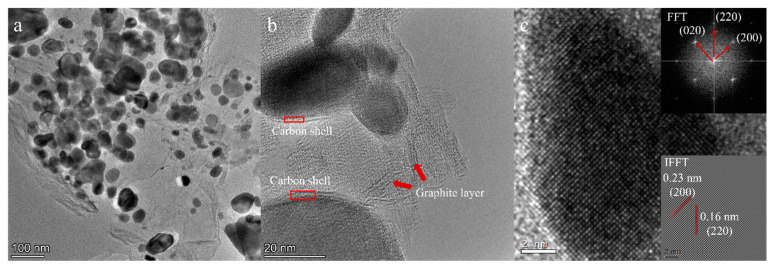
TEM micrographs of R16-1400. (**a**) Overview of the nanoparticles of HfC_x_N_1−x_/C. (**b**) The core–shell structure of a single nanoparticle. (**c**) HRTEM images showing the HfC_x_N_1−x_ phase with SiC matrix.

To assess the morphological characterization and microstructure formed at high temperatures, TEM was used to study the R16 sample pyrolyzed at different temperatures. After annealing at 1400 °C, the sample of R16 shows that the HfC_x_N_1−x_ precipitation is distributed uniformly within the C matrix with a size of approximately 60 nm in Figure 9a. The aggregates are composed of nanosized core–shell particles. The core consists of a single crystal of HfC_x_N_1−x_ and the shell consists of amorphous carbon. In addition, a close examination of the TEM reveals that some graphite-like carbon ribbons are visible in a C matrix in Figure 9b. In Figure 9c, the high-resolution TEM image data demonstrate that the core of HfC_x_N_1−x_ is a single crystal presenting a selected area electron diffraction (SAED) pattern, which can be fully indexed according to the HfC_x_N_1−x_ structure (Fm-3m). In Figure 9d, IFFT results reveal a distinct lattice spacing of 0.16 nm and 0.23 nm, which matches the (220) and (200) planes of HfC_x_N_1−x_.

**Figure 10:**
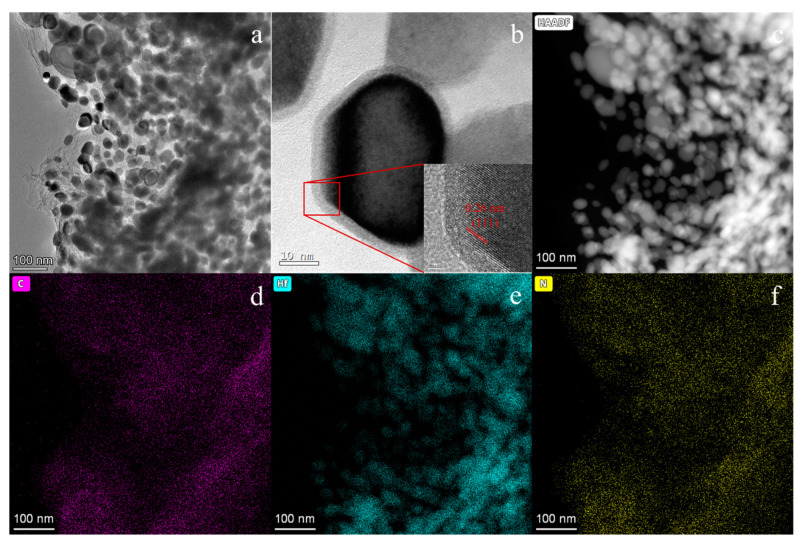
TEM micrographs of R16-1600. (**a**) Overview of the nanoparticles of HfC_x_N_1−x_/C. (**b**) The core–shell structure of a single nanoparticle. (**c**–**f**) High-angle annular dark-field scanning TEM and EDX mapping images of C, Hf, and N showing a homogeneous elemental distribution.

In Figure 10, after annealing at 1600 °C, the nanoparticles remain nanosized. The core–shell structure becomes more pronounced as shown in Figure 10b. The core, consisting of HfC_x_N_1−x_ particles, appears darker in contrast, while the shell, composed of amorphous carbon, appears brighter. The carbon shell thickness is approximately 3–5 nm. In Figure 10b, the high-resolution TEM (HRTEM) image reveals a lattice spacing of 0.26 nm, which corresponds to the crystal plane of HfC (111). This observation confirms the presence of HfC in the nanoparticles. Additionally, the elemental mapping of the nanoparticles in Figure 10c–f demonstrates the homogeneous dispersion of phases and elements, further supporting the presence of HfC_x_N_1−x_ and carbon (C) in nanoparticles. This approach to fabricating nanoparticles shows promising results.

**Figure 11:**
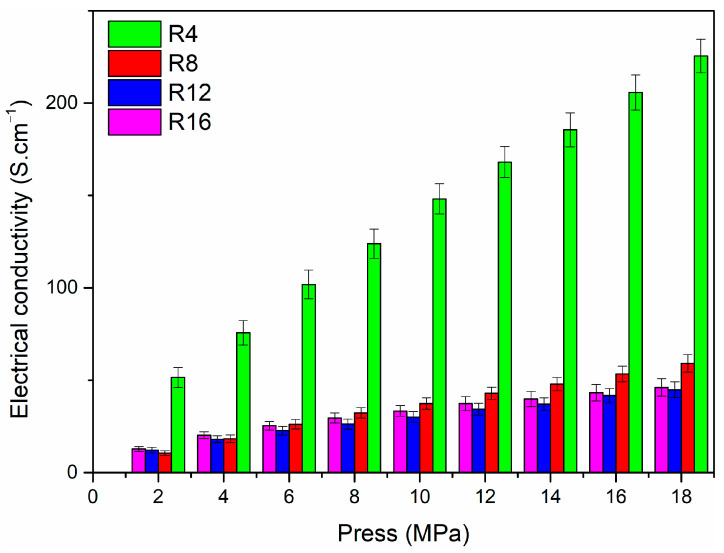
Electrical conductivity of HfC_x_N_1−x_/C nanoparticles with different pressures derived from precursors.

Figure 11 shows the average σDC values for different pressure measured on powders of HfC_x_N_1−x_ ceramic nanoparticles using a four-point probe method. The average electrical conductivity of R4-1600, R8-1600, R12-1600, and R16-1600 nanoparticles with a pressure of 2 MPa is 51.5, 10.5, 12.1, and 12.8 S·cm^−1^, respectively. With the pressure increasing to 18 MPa, these values rise to 225.5, 59.1, 44.8, and 46.0 S·cm^−1^. According to the literature, the electrical conductivity of HfC_x_N_1−x_ is σ = 3~4 × 10^4^ S·cm^−1^ [27]. The σDC of HfC_x_N_1−x_/C nanoparticles significantly decreases followed by a slight increase with the increase in R. It is also interesting to note that conductivity increases significantly with pressure. These trends can be attributed to the following factors. As the pressure on the powder increases, the porosity decreases, increasing electrical conductivity. The TEM results show an obvious core–shell structure with HfC forming the core and amorphous carbon forming the shell. As reported in previous studies, due to the low σ_DC_ of disordered carbon (=1 S·cm^−1^) [27], the amorphous matrix and carbon shell acted as a barrier for electrical transport, which reduced the electrical conductivity of HfC_x_N_1−x_-carbon core–shell nanoparticles. According to the elemental analysis in Table 1, with increasing R, the content of carbon increases. As a result, the HfC_x_N_1−x_ core is encapsulated by a highly disordered carbon shell with low electrical conductivity, which greatly determines the overall electrical conductivity of the core–shell nanoparticles.

## 4. Conclusions

The core–shell nanoparticles of HfC_x_N_1−x_-carbon have been successfully fabricated using a precursor formed by hafnium, urea, and methanol. The atomic ratio of N in HfC_x_N_1−x_-carbon nanoparticles could be controlled by the annealing temperature and nitrogen source ratio. The decrease in nitrogen source or the increase in temperature will lead to a decrease in nitrogen content in HfC_x_N_1−x_. After annealing at 1600 °C, the composition estimated by Vegard’s law from the XRD pattern is HfC_0.90_N_0.10_, HfC_0.86_N_0.14_, HfC_0.77_N_0.23_, and HfC_0.68_N_0.32_, respectively. When the molar ratio of urea/HfCl_4_ was 16, HfC_x_N_1−x_ nanoparticles first appeared at 1200 °C, without the existence of an oxidation phase. As a result, by increasing nitrogen sources, the amount of nitrogen in HfC_x_N_1−x_ will increase, and the carbothermal reaction between HfO_2_ and carbon will change into a carbothermal reaction between HfN and carbon. Due to the low σ_DC_ of disordered carbon, the amorphous matrix and the carbon shell act as barriers to electrical transport, which reduces the electrical conductivity of HfC_x_N_1−x_-carbon core–shell nanoparticles. In addition, the average electrical conductivity of R4-1600, R8-1600, R12-1600, and R16-1600 nanoparticles with a pressure of 18 MP is 225.5, 59.1, 44.8, and 46.0 S·cm^−1^, respectively. The unique HfC_x_N_1−x_-carbon core-shell nanostructure could be a promising material for potential electromagnetic applications in harsh environments.

## Figures and Tables

**Table 1:** The raw material formulation of each precursor.

Precursor	Urea (mol)	HfCl_4_ (mol)	Methanol (mL)
R4	0.04	0.01	20
R8	0.08	0.01	20
R12	0.12	0.01	20
R16	0.16	0.01	20

**Table 2:** Lattice parameters and the estimated compositions of HfC_x_N_1−x_ in nanoparticles at different temperatures are reported, as well as annealed elemental analysis.

Sample	Lattice Parameters	HfC_x_N_1−x_	Estimated Grain Size	Elemental Analysis (wt%)	
				C	N
R4-1400	0.45894	HfC_0.57_N_0.43_	58.7 nm	n.d.	n.d.
R4-1600	0.46264	HfC_0.90_N_0.10_	25.6 nm	5.48	2.0
R8-1400	0.45824	HfC_0.51_N_0.49_	47.7 nm	n.d.	n.d.
R8-1600	0.46224	HfC_0.86_N_0.14_	46.5 nm	9.89	1.08
R12-1400	0.45728	HfC_0.42_N_0.58_	63.6 nm	n.d.	n.d.
R12-1600	0.46119	HfC_0.77_N_0.23_	38.2 nm	14.9	1.01
R16-1200	0.45467	HfC_0.19_N_0.81_	41.5 nm	n.d.	n.d.
R16-1400	0.45720	HfC_0.41_N_0.59_	72.2 nm	n.d.	n.d.
R16-1600	0.46013	HfC_0.68_N_0.32_	45.5 nm	19.3	0.83

n.d. not determined.
